# University of Pennsylvania 11th annual conference on statistical issues in clinical trials: Estimands, missing data and sensitivity analysis (afternoon panel session)

**DOI:** 10.1177/1740774519853565

**Published:** 2019-06-27

**Authors:** Roderick J Little, Eric J Tchetgen Tchetgen, Andrea B. Troxel

**Affiliations:** 1 University of Michigan School of Public Health; 2 Wharton School, University of Pennsylvania; 3 New York University Langone Health

## Roderick J. Little

There has been quite a bit of discussion about the National Academy of Sciences (NAS) study.^1^ I think the main focus of that study was on internal validity rather than external validity, although external validity is clearly important. Key points of the study are as follows. (1) NAS Study Panel defined data as missing if the missingness hides quantities that are meaningful for analysis. (2) The clinical trialists on the NAS Panel made the point very strongly that the best solution to missing data is to design and implement the study in a way that limits to the degree possible the amount of missing data. The reason is that any analysis method for handling missing data comes with unverifiable assumptions, and in confirmatory trials we want to limit those kinds of assumptions. (3) The message from the NAS Panel to prevent missing data has had some impact on practice, with pharmaceutical companies paying far more attention to avoiding it in the conduct of trials. (4) Analysis methods need to be driven by plausible scientific assumptions. If you look at analysis methods in this conference, it is clear that we’ve come a long way from “last observation carried forward” imputation. I think that is good news. (5) The NAS Panel recommended sensitivity analyses to assess impact of alternative assumptions about missing data—I’ll say a little bit about this later. Sensitivity analyses make pharmaceutical companies nervous, but concerns about lack of robustness of findings to sensitivity analysis reinforce the idea that it is important not to have too much missing data, because a large amount of missing data tends to reduce the robustness of a treatment effect.

Let me spend a little time focusing on the estimand, which the NAS Panel viewed as a key feature of the problem. Alternative choices of estimand may have very important implications for the amount of missing data. A slightly less than optimal estimand might be worth adopting if it results in a lot less missing data than an optimal estimand.

A particular form of estimand that reduces the amount of missing data is what I call an “on-treatment summary,” as discussed by Little and Kang.^2^ Dr Mehrotra also mentioned this idea, although he used a different name. Rather than looking at the effect of the drug in a fixed time period, regardless of whether the drug was taken throughout that period, an on-treatment summary measures the effect of the drug using only measures while the drug is being taken, perhaps in a way that penalizes treatment discontinuation. This might be preferable to making up stories about what might have happened counterfactually if you had stayed on a treatment that has been prematurely discontinued. On-treatment summaries do not make sense in some situations, such as survival in cancer treatment studies, but may be reasonable for studies of pain medications, for example. A simple on-treatment summary treats treatment discontinuation as treatment failure; another, for pain treatments, is “area under the curve” for reductions in pain.

In Dr Mehrotra’s excellent example concerning diabetes, he talked about the estimand change in HbA1c from baseline to 24 weeks. That estimand requires assumptions (often not very satisfactory) about what happened to people who discontinued a treatment before the 24 weeks were concluded. An alternative on-treatment summary is the proportion of the 24 weeks when the treatment was being taken and the HbA1c was under control. Discontinuing early reduces this measure of effectiveness, and the measure eliminates the need to impute after discontinuation. Maybe you want to penalize people that discontinue early more than this measure, and such modifications can be agreed on as part of the study protocol. There may be better measures; I am not an expert on diabetes measures, but this example gives the basic idea.

The NAS Panel discussed the following: (1) the appropriate definition of missing data; (2) inference about an appropriate and well-defined causal estimand—like Dr Scharfstein, I would include “causal” in the “estimand” language; (3) the need to document to the degree possible reasons for missing data, and to incorporate this information into the analysis, since some reasons may be plausibly missing at random (MAR) and other reasons may not be MAR; (4) the need to decide on a primary set of assumptions and conduct a statistically valid analysis that takes into account the uncertainty from missing data. The NAS Panel favored likelihood-based methods or augmented inverse probability-weighted estimation over other methods; and (5) the need to assess robustness using a sensitivity analysis.

Sensitivity analysis is indicated because missing not at random (MNAR) models cannot be reliably estimated, suggesting varying the parameters that you can’t estimate in a sensitivity analysis. In many but not all situations, it would be sensible to assume MAR as the primary model and then consider MNAR deviations from MAR in the sensitivity analysis. Two common classes of MNAR models are selection models and pattern-mixture models; I like the pattern-mixture model factorization because I think it’s easier to understand; parameters in selection models are quite complicated to explain to non-statisticians.

An illustration of a sensitivity analysis based on a pattern-mixture model is given by Little et al.^3^ The analysis was for a large trial for assessing rivaroxaban for patients with acute coronary syndrome. About 15,000 patients were randomized into three treatment groups, two doses of rivaroxaban and a placebo, and the primary analysis was by the Cox proportional hazards model. This analysis showed a statistically significant reduction in the primary efficacy outcome, which was a composite of cardiovascular death, myocardial infarction and stroke for the combined rivaroxaban doses compared to placebo (hazard ratio (HR) and 95% confidence interval (CI) = 0.84 (0.74–0.96)). There were concerns about dropouts in this trial, perhaps motivated somewhat by the National Academy study. What if dropouts had worse than expected outcomes, and this biases the treatment comparison? So we did a sensitivity analysis to assess the impact of deviations from “non-informative” or “coarsened not at random” censoring on treatment comparisons.

Since we are focused here on estimands, one interesting feature of this analysis was that there were two estimands that the Food and Drug Administration (FDA) agreed to consider—a strict intention-to-treat (ITT) estimand, which included all events that occurred in randomized subjects until the end of the study, and a modified ITT estimand, which only considered events in the month after the dropout, arguing that the effect of the drug would wash out after 30 days. To editorialize a bit, I think the FDA agreed to consider the modified ITT estimand, but the statisticians thought it was a bit fishy and really favored strict ITT. The interesting aspect for our discussion is that these alternative estimands have very different implications for the amount of missing data: there are a lot less missing data for the modified ITT estimand than for the strict ITT estimand. Statisticians love ITT because of the benefits of randomization, but modified ITT might be a good alternative if one is interested in limiting missing data.

In the sensitivity analysis, we first estimated the hazard for each individual at the time of dropout under the coarsened at random Cox model analysis, but then differentially increased the hazard of the outcome in the rivaroxaban treatment groups, but not in the control group. Then events after dropout are multiply imputed, assuming a Weibull distribution for time to event. Results are combined using multiple imputation combining rules, and then the *tipping point* is found, namely the increase in hazard in the treatment groups at which the statistical significance of the treatment effect is lost, at the 5% significance level. For the modified ITT analysis, this tipping point is 2300%, which is very high. The reason is that there are very little missing data for this estimand. For the strict ITT analysis, the tipping point is 160%, a much lower value because there are a lot more missing data being imputed. This example illustrates that different choices of estimand can differ greatly in the amount of missing data.

So, sensitivity analysis is a good idea, but deciding how to implement it in a regulatory setting is challenging. I think the draft Addendum is a good step forward in this regard. My main quibble concerns the horrible term “intercurrent,” which is not a real English word and lacks a clear logic—how can something that is “current” be “inter”? I would suggest “intervening” rather than “intercurrent,” which is a real and meaningful English word.

To summarize, sensitivity analysis is important; choice of estimand is important and requires thought; and consider estimands that limit the amount of missing data, such as on-treatment summaries or the modified ITT estimand in my example.

## Eric J Tchetgen Tchetgen

I come from the causal inference literature, and missing data literature, and in that literature, people are very, very skeptical. Historically, the way the literature developed is following the road map that’s now being followed by these guidelines, which is start by defining what you’re interested in, define the assumptions under which you’re going to identify it from the observed data, define the main estimators for it or whatever procedure you’re going to use and then see how robust your assumptions might be from possible deviations using some type of sensitivity analysis or an alternative set of identifying assumptions. It’s a welcome move to see these concepts also entering the area of experimental designs or imperfect experimental designs. I’m going to make a few comments about some of the talks today. My level of discomfort with Dr Ibrahim’s talk was about interpretation of the causal estimand. There is a paper by Miguel Hernán^4^ titled “The hazards of hazard ratios” and I think it has implications for some of this work. Finally, I want to discuss an aspect of the issues that have been discussed today that I think has not been addressed, which goes back to what has been said about avoiding missing data. I like to think, as in experiment design, there is an additional ability to be able to use design-based methods to address missing data conceptually.

**Figure 1.**
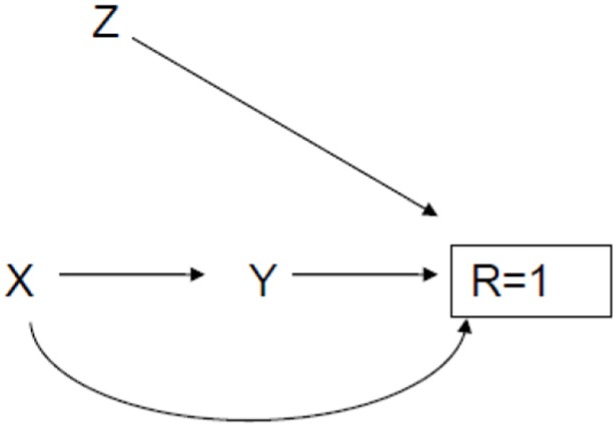
Causal diagram illustrating an instrumental variable.

What I want to discuss is the idea of an instrumental variable (IV) for missing data. It’s a lovely idea, but it’s one that’s not often seen in biostatistics. It’s very popular in the social sciences. A valid IV in the context of missing outcome data is a variable that must not directly influence the outcome of interest in the underlying population conditional on fully observed covariates, and it also has to influence the missingness mechanism conditional on possibly fully observed covariates. Therefore, a valid IV must predict a person’s propensity to have an observed outcome without directly influencing the outcome of interest. Figure 1 shows what an IV looks like.

X, in Figure 1, in the randomized context would be the randomized treatment. Y is the outcome of interest. R = 1 means one has observed the outcome of interest for a given person, while R = 0 means the outcome is missing for the person. There is no direct arrow between Z and Y, the outcome variable. The mean of Y in this graph is a counterfactual estimate in the sense that it’s the mean of the outcome if R were somehow set to 1 for all participants. It’s not directly estimable, but with an IV labeled Z, one can actually improve the level of evidence. Z may in fact be some kind of incentive to either retain or improve participation in your study. In reality, most incentives are imperfect, and so it turns out you actually could randomize incentives, and this could be done within the treatment arms of your randomized trial, and then you have this new experimental design that’s targeting the missing data process, provided it satisfies the aforementioned untestable condition about independence between the incentive and the outcome Y in the population. If randomization is not possible, researchers could still carefully select observational IVs for modeling missingness.

An example is provided by an observational study from Zambia where they were trying to estimate HIV prevalence in men. The study personnel went to 7164 households and 5145 provided a specimen for testing, so they had about 30% missing outcome data in the sample. They had interviewer characteristics, age, gender, years of experience and the language of the interviewer who was dispatched to the household to ask people to volunteer to test. And that, in fact, turns out to be a very strong IV in this context because one can assess the correlation between interviewer identity and the nonresponse rate. However, there is an untestable assumption here, which is that interviewer characteristic does not directly affect the outcome of HIV prevalence, which is what you would expect because there is no biological reason for a direct effect to be present. However, such an association may be present and therefore the assumption violated, if, for instance, you have areas where it’s harder to get a good response (i.e. agreement to test for HIV) rate, which also happens to also have a higher rate of HIV associated with higher risk behaviors. You might dispatch your best interviewers, to such areas and so in those settings you have to be careful because interviewer characteristic would likely be associated with HIV rate. In the Zambia study, interviewers were dispatched at random, so there was no such concern. Given the IV design, you can test for and assess the impact of increasing departures from the MAR assumption. You can construct bounds for the underlying parameter of interest; these are bounds that have been in the literature for a while. For inference, you have to be a little careful because there are max and min functions involved in defining the bounds, which renders the bound a non-regular parameter; however, methods are available that can deal with this possible complication. And just to show you how these bounds work in the Zambia example, the complete case analysis estimate was 12.2% HIV prevalence. Applying the IV information, you get an almost twofold increase in the estimated prevalence of HIV in Zambia along with bounds that capture uncertainty in the stated assumptions (21.1% (95% CI = 16.2%–25.9%)). In summary, I like the idea of trying to use design-based methods for retention and overlay those on your original design.

## Andrea B Troxel

The concept that prevention is critical is not a new idea, but it’s helpful to restate, and restate often. Sensitivity analyses are also critically important. And a theme of the day has been that there is no substitute for very careful thought. Going back to the very first introduction this morning about the ICH Guidance document and its revision, the primary, really critical message of that document and the ideas behind it is that we really have to think hard about what we’re doing and how to interpret what we’re doing. Dr Scharfstein gave us a very interesting and thoughtful presentation, and what I took away from that largely is that this idea of treatment strategies is really important. The idea that they are definable, and include contingency plans for the various things that might happen, I find really useful. We probably can’t predict in advance all of the possible contingencies that might come up, but we can predict many of them, and so we should build that into our plans. Also, this issue of defining adherence very precisely is something that I’ve given a lot of thought to in the context of behavioral trials (that I’ll describe in a minute) and I think that’s also something we have to focus hard on. Last, Dr Ibrahim’s talk was really a nice example of important methods development that arose from a real clinical problem. He gave us a nice example of treatment switching in a colon cancer trial, which is, as he described, something that happens all the time in this kind of framework. We can argue about the interpretation of the time-averaged parameters that he has shown us how to estimate, but the concept of building all of that in from the beginning and really thinking hard about it, is a theme that we’re focusing on.

There are many directions that we can go in terms of moving forward. I want to propose that we think about additional context for these kinds of thoughts and principles. The original Guidance and the revision of the document were developed squarely in the realm of drug trials, in which the issues of treatment adherence are, if not easily managed, as least fairly well-defined. I have done a lot of work of late in settings involving behavioral interventions that differ substantially from drug interventions. And in that context we can learn a lot from the kind of thinking that is going on within the context of drug trials, as well as vice versa. Some of the issues that we grapple with in these less well-defined behavioral settings might also inform some of what we think about in the setting of drug trials. In the interest of time I want to give just one example, a trial that I worked on with colleagues here at Penn. This is the Shared Incentives Trial that was led by Drs Kevin Volpp and David Asch, who are both general internists here. It was a cluster-randomized trial involving several hundred physicians in three different health systems, with 1500 participants, and pretty broad eligibility criteria. Participants had to have high cardiac risk and elevated low-density lipoprotein (LDL) cholesterol. The goal of the trial was to reduce LDL cholesterol in participants; change in LDL over a year was the defined primary outcome, and there were four interventions. I tend to use the word “intervention” rather than “treatment” or “drug” because most of my interventions are not drugs. There was a control arm. There was an arm in which patients received daily incentives to adhere to their statin medication (with a daily lottery for statin adherence). There was a physician incentives arm in which the physicians received direct payments if their patients achieved certain reductions in LDL cholesterol. And there was a shared incentives arm in which both the patients and the physicians received the incentives, but each at half value, so that the total expected value of the incentives was approximately equivalent.

**Figure 2.**
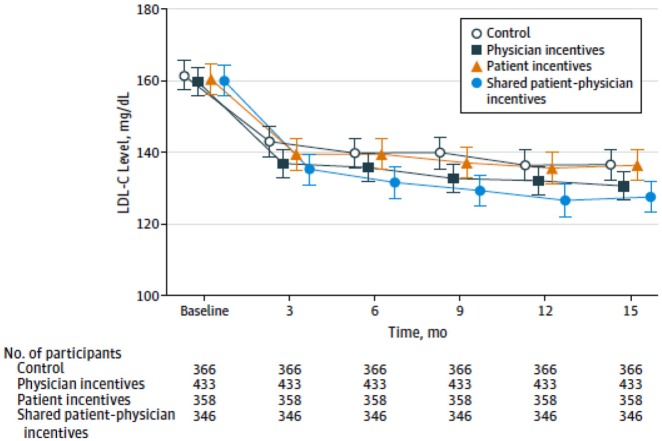
Mean LDL-C levels by quarter in intervention and control groups. To convert low-density lipoprotein cholesterol (LDL-C) to mmol/L, multiply by 0.0259. Error bars indicate 95% CIs (with permission).^5^

The results of the trial are shown in Figure 2. This is a very interesting picture and not exactly what we expected to see when we designed this trial. You can see that all of the patients in all of the arms showed substantial early reductions in their LDL. Most of those reductions occurred in the first 3 months, with pretty steady maintenance of those reductions over the course of the trial. The shared incentives arm had the greatest reduction, but even the control arm, as you can see, did surprisingly well. This is an ITT estimate. During this trial we (luckily) tried to follow Dr Little’s advice and prevented a large amount of our missing data; we had an incredibly dedicated team of research coordinators and staff who did an amazing job, and we ended up having only about 10% missing data over the whole 15 months of observation time. Thus, the treatment of the missing data itself did not have a major impact on the results. But the ITT estimate here estimates exactly what we want to know. This is the case in many of these kinds of interventions where the question really is “what is the impact of this program on the outcome of interest in this patient population if this program were offered?” That is something that insurers and payers and health systems and other organizations really want to learn. These interventions are supplementary in some sense to the primary treatment, which is to take a statin. We know from many studies long ago that statins are effective in reducing cholesterol with relatively manageable side effects, and so it should be easy to just give patients statins, and they should do better. But we also know that 30% of prescriptions go unfilled; 50% of patients who start taking a statin within a year don’t take it anymore. There are lots of reasons for that, and many possible responses, but this intervention was an attempt to see whether we can influence adherence, and in turn, achieve the desired change in cholesterol.

**Figure 3.**
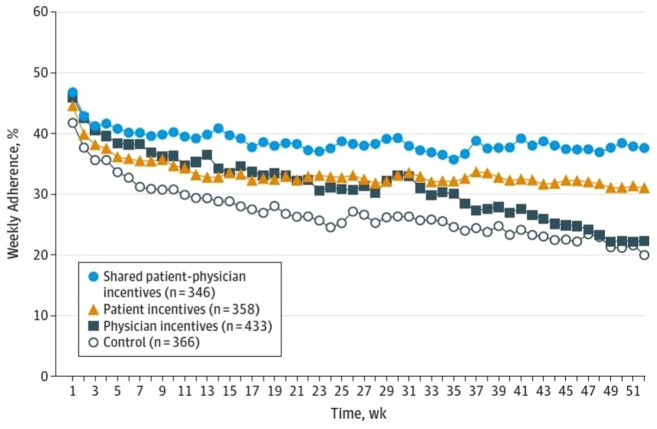
Mean weekly medication adherence by intervention group. Adherence was calculated by dividing the number of pill bottle openings per week by 7. Standard deviation for the shared patient–physician incentives group was 1.8%; patient incentives, 2.4%; physician incentives, 5.5%; and control, 4.4% (with permission).^5^

How could we explain the result that only the joint incentive had a significant impact? Well, there are two intermediate outcomes that we measured. One is adherence, which was measured by electronic pill bottles so that we knew when the patients were opening their bottles, and the other was incidence of either a new prescription for statins or intensification of the statin, either increasing dose or a change to a more potent medicine. There are some interesting stories there as well. Figure 3 shows the average adherence over time as measured with the electronic pill caps, and you can see that indeed the shared incentive arm had the highest adherence. The control arm had the lowest adherence, and the two arms in which patients received incentives had significantly greater adherence than the control arm; in contrast, the patients in the arm in which only physicians received incentives, perhaps not surprisingly, did not have greater adherence than the control arm. I don’t have a plot for the intensification or prescription activities, but we found, again, what in retrospect seems obvious: that in the shared incentive arm and in the physician incentive arm, there was a statistically significantly higher rate of intensification, and that did not occur in the patient incentive arm and the control arm. So to summarize, physician incentives alone are no better than control. Patient incentives alone are no better than control. Shared incentives are better than control even when each is given at half value. My colleague Dr David Asch likes to say that it’s a strange result in chemistry when you combine two inert substances at half potency and achieve an effect—but that’s what we found here. And adherence generally was really disappointingly low. If you look at the y-axis on this plot, you can see that it’s less than 50% for almost the entire duration of the trial. So what’s going on there? Well, again, in retrospect, two things have to happen for the statin to get into the body of the patient and therefore have its intended effect of lowering cholesterol. The patients can’t take medications that they don’t have prescribed for them by the physician, and the physician can’t influence very effectively the patient to take medications even when they’re prescribed. And so the combination of those two things is really critical.

This trial, then, is like a drug trial and not like a drug trial at the same time. The interventions were not connected to anything relating to prescription or administration of drugs to the patients. The interventions were based on behavioral economic principles that we’re trying to leverage to help people make the right decisions and adhere to their medications. The issue of adherence, in this trial, is very interesting. We can measure adherence to the statin medication, which is the purported mechanism by which we expect that the cholesterol will in fact be reduced, but what does adherence to the *intervention* mean? From the patient perspective, the patient incentives were in the form of a daily lottery, so maybe adherence means that the patient remains engaged, pays attention to the lottery messages and opens the pill cap. And what is adherence for a physician who’s receiving payments? We thought that the best way to implement this incentive was to let the physicians manage on their own—they’re being incentivized to reduce LDL in patients better than they had been doing, but how they want to do that is really up to them, and we didn’t want to be prescriptive. Some physicians hired a nurse manager to follow the patients more closely. Some physicians said to their existing operations managers, “if you can manage to do this and get our patients to reduce their cholesterol, I’m going to split the incentive with you.” They did a lot of different creative things, and they did a lot more creative things on their own than we may have been able to predict and plan in advance. Regardless, it’s very difficult to define adherence in this context. In thinking about what adherence is, but also in thinking about what treatment strategies might look like, even in the context of drug trials, developing treatment strategies that incorporate expected and potentially unexpected contingencies is very useful. In the context of some of these behavioral interventions, understanding the different ways in which these behavioral tools might be used is also useful to think about and rewards careful planning.

Again, a theme of the day is that there’s no substitute for planning, and the more you consider ahead of time, the more likely you are to be able to manage whatever it is that actually happens.

To wrap up, as we’ve been repeating all day, clearer specifications are really critical, and that applies to many features of trials. That applies to the interventions that are used and whether those are interventions like that ones I’ve described or treatment strategies that are defined. It applies to the outcomes, to the hypotheses that we’re trying to test, and to the issue of the target population. I spend a lot of time thinking about pragmatic trials and the degree to which standard controlled trials and pragmatic trials estimate different things, and I think that’s important to keep in mind. And to conclude, I think we have a moral imperative to get this right. We’re all here because we care about improving health: health in different populations and health in different aspects. We do our research, and we come up with results, and they get out in the world, and people make decisions as a result; it matters to populations, and it matters to individuals. And so we really have to keep in mind that we have a substantial responsibility to think about our research as carefully and as honestly as we can and then try to make as much progress as we can.

## Mary E Putt

Dr Troxel presented a behavioral trial of treatment for lipid control. The control arm, without the use of incentives, had better lipid control than expected. I’ve seen this type of result in other similar studies. I think we might hypothesize that we have such great research teams that the control itself, as we’ve been trying to reduce the amount of missing data, has actually wound up being a pretty substantial intervention. There are a number of possible explanations for this, including the idea that contact with the research coordinators is an incentive to be medication adherent—even in the control group. Can you comment?

## Andrea B Troxel

Part of the explanation for the dramatic improvement in the control group in the particular trial I gave as an example is this. All patients received the Vitality^®^ GlowCap^®^ electronic pill bottle which electronically reported the opening of the bottle. However, it really wasn’t a true control—it wasn’t usual care. Most people don’t receive these cute electronic pill bottles. They were in so-called “coma mode,” which means they didn’t light up and had no reminder bells; they were designed to look like a regular prescription bottle. But the patients knew that we would be aware of their pill bottle openings. And interestingly, at the end of the study, we learned from the patients in the control group that they liked the bottles—they felt that we were watching over them in a benevolent way, which was encouraging. They didn’t think it was “Big Brotherly,” which is something we were a little concerned that they might. But they knew that we were aware of their information, and they felt that they were being provided some level of extra care in a way that they wouldn’t ordinarily have been. And so it seems very likely that if that was the mindset of the so-called control patients, then in fact the provision of what was meant to be an “inert” bottle clearly was an intervention of some kind, and they responded to that. There were some other things that happened within the health system simultaneously, somewhat unluckily for us in terms of the trial perspective, with efforts to improve cholesterol control in general that were concurrent with our trial; that was an accident of circumstances, but also probably contributed to the good outcomes in the control group. But this issue is a big one, and in the world of behavioral interventions this phenomenon of a so-called “attention control”^6,7^ is something that is important and useful to consider: if part of your intervention is that you’re interacting with somebody on a daily basis, maybe it’s just that interaction that’s effective, unrelated to the substance of the intervention. Those two are often conflated, and thus a lot of careful design thinking needs to go into not only design of the trial in the usual statistical sense but design of the intervention to understand what the various active components might actually be.

## Samiran Ghosh

The avoidance of missing data, though attractive, is impossible to achieve in many clinical trials. The reasons for missingness, if they can be identified along with other trial data, could provide great insight in differentiating avoidable versus non-avoidable reasons. This in turn can reduce the amount of missing data and give additional data and methods for adjusting for the potential bias. One technique developed in mental health by Leon et al.^8^ is called Intent to Dropout. Basically what they proposed in a longitudinal trial is that every time a patient is being evaluated, he or she is given a short survey, with questions that capture patients’ intent to continual participation in the trial. This survey/scale can be used in estimating the chance that the patient will not come back to the next visit. So they proposed that if the probability of not coming back is more than a certain percent, let’s say 70% or 80%, then the investigator can make a tailored approach to see whether missing the next visit is preventable. Of course all missingness is not preventable, but this approach could give us a framework for bias reduction with an adjustment for participants’ intent to dropout. I would like to know whether this can be done in many clinical trials and what are pros-cons of similar approach.

## Roderick J Little

I think it’s a reasonable idea.

## Andrea B Troxel

I think it’s a very useful idea. In practice, it’s very difficult because it’s hard to measure those sorts of propensities, and also because of the social desirability bias it’s often hard to get people (either patients or clinicians caring for patients) to admit that they’re likely a flight risk in some sense. Conceptually it makes a lot of sense, but I fear that practically it may be quite difficult.

## William Wang

I think the estimand in the survival analysis setting is intriguing, although in this setting we use the term censoring, not missing. I was very interested in whether the hazard itself is the right measurement for the causal estimand or is there an alternative way to measure in the causal estimand framework in the survivor setting? Also, in the survival analysis setting, are the techniques in some way similar to the principal stratification framework?

## Eric J Tchetgen Tchetgen

There are methods other than principal stratification, for example, to deal with treatment switching problems in the survival context. One that has been very successful, and actually I think has been applied a lot in the context of cancer research, is a structural accelerated failure time model. And it really treats both the intervention of interest and the secondary intervention, the salvage therapy, whatever you might call it, as a joint intervention, and it asks the question, if I were to actually intervene to prevent anyone from taking the salvage therapy, what would be the direct effect of the intervention of interest on the endpoint, the time to event outcome. So that’s one set of methods that’s appealing because it actually leverages randomization to estimate the effect of interest.

An alternative set of methods based on inverse pattern weighting of marginal structural models can also be done in the Cox setting, and there are published papers that describe them at length. They tend to rely on the assumption that you’ve measured post-randomization covariates that might confound the salvage therapy, so they rely on stronger assumptions, but they are all laid out in the papers, and there are ways to assess those assumptions in the sensitivity analysis. And then finally, I wanted to say, the issue with the HR, as noted by Miguel Hernán is about the built-in selection bias in the hazard function. The hazard at a given time point is conditioned on having survived up to that time point, which is post-randomization, and so the interpretation of the HR is shaky. It has a built-in selection bias, and so people have lately tried to move away from it. I understand that’s kind of the standard in randomized clinical trials, and the FDA expects to see HRs. I’m just telling you this is where the literature is going.

## Daniel O Scharfstein

I think a lot of the confusion that we have in some of these questions can be solved by really understanding the framework of causal inference and potential outcomes; I encourage you to download Causal Inference by Hernán and Robins.^9^ It’s a very readable volume. Once you start thinking about survival analysis in the context of potential outcomes (e.g. time to event under treatment, time to event under control), you realize what the problem is with using Cox proportional hazards models to estimate the intent-to treat-effect in randomized trials. I just think a lot of the misunderstandings (and my disappointment with the Addendum) result from avoiding the causal inference framework. I think a lot of the confusion would go away if there was more understanding of the potential outcome framework.

## Zhehui Luo

I have a question for Dr Tchetgen. This morning you made a comment that sounds like the principal stratum estimand is not ready to go prime time, but this afternoon you gave an example where you used the interpersonal skills of the interviewer as an instrument for missing data. I supposed that’s not the primary aim of that trial because it’s not built into the design stage, so how do you define the estimand in that situation? Can you give some more thoughts about your morning and afternoon contradictory comments?

## Eric J Tchetgen Tchetgen

I don’t think there’s a contradiction. I think what I talked about this morning is what Dr Scharfstein talked about as well, which is that principal strata are defined in terms of the joint distribution of potential outcomes. Consider Dr Mehrotra’s example, the effect of vaccine on viral load among individuals who would be infected with HIV irrespective of whether or not they received the vaccine. You have no idea who those individuals are. You have no idea who they are in the next population. Often behavior changes after a trial result comes out. Who knows? I’m mixing up examples here because you had a question about compliers, which is also another form of principal strata which is less problematic as far as I’m concerned because in some settings, like a randomized trial where the control arm doesn’t have access to the active treatment, with reasonable assumptions under randomization and double blinding, you can identify the complier causal effect.

## Devan V Mehrotra

Two things. First, I want to come to the defense of principal stratification since we have used it as a primary estimand strategy with a corresponding pre-specified primary analysis in a landmark clinical trial, and so I get very uncomfortable when I hear suggestions that the moment you hear principal stratification just turn around and run. But imagine the situation that we were in, where the vaccine being developed was less likely to prevent HIV infection but more likely, because of the mechanism, to keep the viral load set point at a manageable level among those that became infected. In the best case scenario, the infectees would not require any antiretroviral medication because the immune system would have been augmented enough to keep the viral load set point at a very low level. I would submit that principal stratification is the only strategy that you can use in that scenario. So while I accept the notion that there are challenges and you have to be careful about it, I worry some people might walk away from today’s conference inferring that the principal stratification estimand strategy should not be considered. And second, there’s been discussion about Miguel Hernán’s recent work about whether, under proportional hazards, an HR has a causal interpretation. Is it a “causal estimand”? I would submit the answer is yes, depending on how you define a causal estimand. If you insist that a causal estimand can only be defined as a population summary of a within-subject comparison of treatment A to treatment B, then a difference in medians cannot represent a causal estimand. So ultimately, if you’re willing to accept an alternative definition of a casual estimand that contrasts a meaningful population-level parameter under treatment A to the corresponding parameter under treatment B, then an HR would qualify as a causal estimand under a proportional hazards assumption because the ratio of hazard functions would be time-invariant.

## Eric J Tchetgen Tchetgen

I agree with Dr Mehrotra. Maybe I came out too strong, but what I did say this morning was in response to a question that was asked about whether principal strata should be used for regulatory purposes. I will stand by that part of what I said, which was I think it should be understood as purely observational—I mean a purely explanatory analysis and not an analysis that I would feel uncomfortable with guiding policy. It’s not about something that could ever be needed by any experiment. Principal strata are not identifiable, and that’s the reason for my discomfort. You define the parameter, you estimate it with the data you have, and that’s great, but maybe that should be used to inform further exploration about whether to approve the drug and for what population, so in that sense, I think it’s a very useful quality. I’ve written about principal strata, I’m not damning it, I just think it’s a different animal altogether than the average causal effect.

A few words about the HR. You might say the HR is a population parameter that contrasts two distributions under treatment or under control. That is true, and that’s how it’s being used primarily. At any given point in time, post-baseline, it does not have a causal interpretation. Why? Because there are two effects. First, conditional on having survived up to that time point you have the effect of the treatment on surviving. And second, you actually have a causal treatment effect on the hazard at that instantaneous point in time. Among these two, the first one can be affected by selection bias. I don’t think we disagree with that, and Miguel Hernán indicates this in his paper. You have to be careful. Is it really the causal quantity that we care about in the context of time varying at-risk populations?

## Roderick J Little

I like the idea of defining causal effects for individuals rather than for populations, but I do think it’s worth making a distinction between fitting a model to the population—to a sample in order to essentially smooth the data—and estimating a causal effect. For example, a coefficient on treatment in logistic regression is not, to my mind, an individual causal effect, but you can fit a logistic regression to a population and then estimate a causal effect from the predictions from that logistic regression. So I think distinguishing the causal effect for the individual from how you model the data is useful.

## Daniel O Scharfstein

I was trying to be provocative today, because I know that the approval process for drugs and devices can be based on precedent. Consider an example where drug company A receives approval based upon primary estimand as being a principal stratification estimand. Now drug company B is going to come along and say, hey, you did that trial, you got your drug approved with that principal strata so I’m going to do that. But the assumptions underlying those analysis can be so incredibly strong—the level of evidence has been reduced to that of an observational study. We don’t typically approve drugs based upon observational data. I’m just worried and feel like the document should have been a bit more cautious. I hope that there is caution throughout the FDA when a drug company comes and says that they want to get this drug approved with a principal stratification estimand that’s been disseminated. As Dr Troxel said, we have a moral imperative, and we’re here to basically ensure that drugs are approved for the good of the public.

## Michael Schell

I also want to follow up on the moral imperative that Andrea pointed out. Most trials and studies that are done are not going to be regulatory studies. Many of us are working on studies that are not done in that environment; they are done a lot less rigorously than the ones that go to the FDA. And so the moral imperative becomes even more important on those kinds of studies. We have been talking about incentives—to incentivize doctors to give what you think is the right medicine, and the patients to take it. What incentives are there for the researchers and so on to do a well-designed study in a non-regulatory environment?

## Andrea B Troxel

We could talk about incentives in a broad sense. I think it’s up to us to do things correctly, thoughtfully and honestly. I think that that goal in and of itself should be enough, and that should be what motivates us to do the work that we do in as high a quality way as we possibly can. I’ve been lucky to work with people who have the same view, and in general, we try very hard. We don’t do everything right, and I’m sure we’ve made mistakes, but we think deeply, we try hard and we do our best. It sounds naïve to say that, perhaps, but that’s why we’re all here and that’s what we all should do. The more systems to support that behavior that we can put in place, the better. We want to make it easy for people to do that high-quality work and think as carefully as possible. Having these guidance documents is actually an important step, because it provides structure, which is very helpful, and it provides standards, which are very helpful. We’re all human, and we want to do things according to how they’re supposed to be done; if we have some authoritative group that is setting standards and expectations for how we do things, that is actually incredibly motivating. I think the fact that we’re all here discussing this is a very positive thing. We’re arguing a little bit and we have some differing views about a lot of details, but everyone is here because we care deeply about insuring that we do the best that we can. I think having communities of academic, industry and regulatory partners work together discussing and talking and working really hard to provide these standards is critical.

## Mary Sammel

I wanted to respond to the comment about why in academia we would want to make sure that we have high-quality research, and I think it’s because we’re going to publish our research. Increasingly the journals are very rigorous about demonstrating that your trial was registered, that your outcomes, as defined a priori, are the ones that are in the paper.

## Roderick J Little

I want to ask Dr Tchetgen a question about IV. I’m a little familiar with these IV surveys where they randomize interviewers, and actually I like that quite a bit. It’s a little bit hard, however. I’m really skeptical about IV variables aside from randomization, which is a very good IV. And it’s hard for me to see how you do that in a clinical trial context. You know, whether you’d find very good IV variables. I mean, you can’t randomize the clinician to the patient, I don’t think, so do you have any ideas?

## Eric J Tchetgen Tchetgen

In the opening example of my presentation, the intent was to randomize incentives first for retention. So for instance, in Botswana what you might do is to encourage people to test for HIV. You might say, you’re going to flip a coin, and depending on the outcome, there’s a good chance that if you participate in the study, you would receive a pre-stated amount of cell phone airtime as compensation.

## Roderick J Little

Would this be differential incentives for getting to the end of a study, for example?

## Eric J Tchetgen Tchetgen

Exactly, and that you could randomize.

## Participants

**Table:** 

Samira Ghosh	
Roderick J Little	Panelist
Zhehui Luo	
Devan V Mehrotra	
Mary E Putt	Moderator
Mary Sammel	
Daniel O Scharfstein	
Michael Schell	
Eric J Tchetgen Tchetgen	Panelist
Andrea B Troxel	Panelist
William Wang	
